# The Visiting Doctor

**Published:** 1919-02

**Authors:** 


### The Visiting Doctor.

For many reasons it's a pity that the age appears to favor the passing of the old-time visiting family doctor. A wonderful man was this once-familiar figure, especially in the country with his well-known and faithful old horse and his "pillbags." His visits were of equal, if not greater moment than those of the circuit rider. He not only carried his "physic" with him, but his stock of good cheer was more comforting than his medicines. He carried hope and optimism wherever he went and when he left a patient the entire family felt that there was something in life worth living for.

The trend now is toward having the patient go to see the physician instead of having the physician go to the patient. The doctor receives his patrons in a handsomely furnished suite of rooms, where there are usually other patients awaiting their turn. Each in his turn, like a barber's customers, goes into the little consultation room and sits in a stiff, unforbidding chair to answer questions or to be examined. The doctor is in a hurry to get through; the patient is nervous, because he knows that others are waiting outside; no time is taken with the little social amenities that once so endeared the physician to every patron; the whole atmosphere smacks of commercialism. Under such conditions the patient cannot feel that the physician has much more interest in him than in any other "case" on the street—that he is merely a business machine treating all who come to him as so much grist.

Not only do the patients fail to get that "consolation" that once was a large part of the stock in trade of the family physician who visited the home, but the doctor does not get that helpful return that once characterized his work. He no longer feels that he is in any sense a sort of missionary going about doing good. The best part of any service is the satisfaction that comes to the one who gives the service,—even the money received, while essential, is not to be compared with that feeling of real helpfulness. The office practitioner does not get that in any large measure.

Then, the office man, who does his work in his office laboratory or in the hospital, fails to get that advantage that comes from an active out-door life. His sedentary occupation in close rooms with ill-smelling drugs, in time takes all the energy and vitality out of him, and he becomes a mere operating machine, the victim in small measure of every case that comes under his treatment. In time the papers report that Doctor So-and-So has become the victim of overwork and has been forced to give up his practice for a time. The truth in most cases of this kind is that the doctor has not overworked, but has under-exercised and has not taken that care of himself that he has demanded of his patients.

It may not be possible to return to the old ways, and it may be that in the hurry and skurry of present day methods this is not to be desired, but the modern physician should at least require himself to take some of the precautions against an early breakdown that he demands of his patients. He would be better off physically and mentally, if not financially, if he would spend at least a part of every day out visiting his patients, instead of devoting all of his time to office work, and he would have a closer place in the hearts of those he serves.

---

It is with unusual pleasure that we welcome the return this month of one of our former advertisers, Fairchild Brothers & Foster. We know that the readers of this journal will be glad to see the products of this firm advertised again in this publication. Notwithstanding the high prices caused by the war Fairchild Brothers & Foster have maintained the high standard of their products, which are high grade now, just as they have always been.

---

Among our old advertisers who have placed their advertising in this journal again, after a brief suspension due to the war, is The Denver Chemical Company, who are well-known to all our readers. This is one of the most reliable firms in the country, and goods put up under its name are known to be all they are represented. Now that the war is over the Denver Chemical Company is prepared to extend its business, and the Journal's readers will be glad to help with their patronage.

---

Owing to the numerous complaints from the Medical men of the difficulty and delay in procuring preparations the Anglo-French Drug Co. Ltd., announce they have now established a depot at 1270 Broadway, New York, N. Y. Henceforth all requests by phone, letter, or messenger will receive their immediate attention. Their three preparations for the treatment of syphilis are known as Ampsalvs, Supsalvs, and Mersalvs. Not only are those effective and reliable, but the manufacturers claim to have yet to receive a complaint of any undesirable result following administration, although they have supplied many hundreds of thousands to all parts of the civilized world.

---

The Editor wishes to call attention to the fact that the article which appeared in last month's Journal on "Appendicitis Complicating Pregnancy," was written by Dr. Anne Paul Heineck of Chicago, not by Dr. Anne Paul, as it appeared on the printed page.

